# Assessing the reliability and validity of the sport motivation scale-II using the COSMIN methodology—a systematic review

**DOI:** 10.3389/fpsyg.2026.1807146

**Published:** 2026-05-21

**Authors:** Yuanye Zhu, Yifan Yang, Weidong Gao, Heng Tian, Peijia Yan, Changnian Zhang

**Affiliations:** 1 School of Dance and Martial Arts, Capital University of Physical Education and Sports, Beijing, China; 2 China Wushu School, Beijing Sport University, Beijing, China; 3 Zhejiang Sports Science Institute, Hangzhou, China; 4 College of Physical Education and Sport Science, Qufu Normal University, Qufu, China

**Keywords:** COSMIN, reliability, SDT, SMS-II, sport motivation, validity

## Abstract

**Introduction:**

The Sport Motivation Scale-II (SMS-II) has been widely employed to assess motivational orientations in athletic populations; however, its measurement properties remain subject to debate. This systematic review aimed to evaluate the measurement properties of SMS-II and synthesize the quality of evidence using the COnsensus-based Standards for the selection of health Measurement INstruments (COSMIN) methodology.

**Methods:**

A comprehensive literature search of PubMed, Web of Science, EMBASE, PsycInfo, and CINAHL databases was conducted up to 20 March 2026 to identify studies reporting psychometric evaluations of SMS-II. Measurement properties were appraised against the COSMIN quality criteria, while methodological rigor was assessed using the COSMIN risk-of-bias checklist.

**Results:**

Moderate-quality evidence supported the sufficiency of content validity (relevance and comprehensibility) and six-factor structural validity (supported by 76.5% of included studies). Furthermore, high-quality evidence from a subset of studies (two studies) supported sufficient five-factor structural validity. High-quality evidence also supported sufficient cross-cultural validity and measurement invariance, internal consistency for the six-factor structure (Identified subscale) and five-factor structure (Integrated and Intrinsic subscales), test–retest reliability (except for the Identified subscale), and hypothesis testing for construct validity. Conversely, two-, three-, and four-factor structural validity solutions, as well as internal consistency for the Identified, External, and Amotivated subscales within the five-factor structure, were rated as insufficient. The remaining measurement properties demonstrated inconsistent findings despite high-quality evidence.

**Discussion:**

While specific structural models (two-, three-, and four-factor) and the internal consistency of certain subscales within the five-factor model received insufficient ratings, the core measurement property of the SMS-II as a whole was classified as inconsistent. These findings indicate that the SMS-II can be cautiously utilized, although additional high-quality research is required to establish the sufficiency of its currently inconsistent measurement properties.

**Systematic review registration:**

https://www.crd.york.ac.uk/PROSPERO/view/CRD420251163217, CRD420251163217.

## 1 Introduction

Regular physical activity and sport participation confer multidimensional health benefits encompassing muscular strength (Goodpaster et al., 2008), body composition (Westerterp et al., 1992), sleep quality (Wang and Boros, 2021), and quality of life (Marquez et al., 2020). Notwithstanding the established health benefits of regular physical activity, abandonment of personal exercise programs remains pervasive (Finne et al., 2019). Consequently, promoting sustained engagement remains a critical challenge for exercise prescription developers, physical education instructors, and coaches. Self-Determination Theory (SDT) emphasizes the importance of motivation (e.g., pleasure, excitement, interest, and fun) in maintaining health behaviors, such as physical activity (Teixeira et al., 2012). Motivation is a fundamental psychological construct that drives the direction, intensity, and persistence of an individual’s behavior (Wlodkowski, 1978). In the sporting context, it is a critical determinant of an athlete’s sustained engagement (Gardner et al., 2017), exercise intensity (Duncan et al., 2010), and ultimate performance outcomes (Alkasasbeh and Akroush, 2025). It plays a significant role in initiating, regulating, and maintaining behavior (Paic et al., 2017). SDT explains that motivation is a “construct that can be ordered on a continuum” (Howard et al., 2017). This theory deals explicitly with the issue of motivation as a multidimensional construct that extends on a continuum from amotivation (when an individual has no desire or intention to participate in an activity) through external motivation (when an individual is active only because of the external value that the activity brings) to intrinsic motivation (participation for satisfaction) (Deci and Ryan, 2000). Motivation is important in many fields, such as online learning (Ferrer et al., 2022), academic achievement (Kriegbaum et al., 2018), wellbeing (Nie et al., 2015), and sports training (de Van Pol and Kavussanu, 2012).

Researchers have long studied motivation in sport to understand why athletes differ in key behavioral outcomes, such as varying rates of training adherence or susceptibility to sport dropout, noting that some persevere through setbacks, while others ultimately give up (Pelletier et al., 2019). Therefore, it is essential to accurately measure the motivation of athletes to participate in training. Motivation, however, is difficult to measure, as it is a subjective and latent variable (Chin et al., 2021). Early studies have attempted to assess motivation through verbal reports (Clancy et al., 2017) and by observing the time that a study participant spent on a specific task (Lepper and Greene, 1975). Consequently, researchers have attempted to develop effective measurement tools to better quantify and determine a person’s motivation in sports and exercise.

In 1995, Pelletier et al. created the original Sport Motivation Scale in two languages based on the SDT framework: English (Pelletier et al., 1995) and French (Brière et al., 1995). Both versions underwent extensive validation. SMS has also been criticized for several issues. For example, the scale did not represent all constructs in the SDT framework (due to the lack of an integrated regulation factor), and the confirmatory factor analysis yielded lower fit indices for the SMS subscales. Therefore, Pelletier et al., based on the original SMS, combined various suggestions and made several modifications, including adding the Integrated subscale, refining the measure of intrinsic motivation, and reducing the number of items per subscale. The revised scale is called the Sport Motivation Scale or Sport Motivation Scale-2 (SMS-II). Since SMS-II’s publication, extensive research has investigated its measurement properties (reliability, validity, and responsiveness). Despite numerous studies reporting adequate psychometric performance, contradictory findings persist. For example, test–retest reliability for the Identified subscale has been classified as insufficient in one study (Intraclass correlation coefficient [ICC] = 0.53) in a sample of adolescent Malaysian athletes (Chin et al., 2021) and sufficient (ICC = 0.79) in other demographic and cultural contexts (Li et al., 2018), suggesting that sample characteristics may introduce substantial variability. Such discrepancies underscore the substantial heterogeneity across investigations, warranting a systematic review with a formal evidence quality assessment. While one study (Amaro et al., 2025) synthesized SMS-II psychometric evidence, their review lacked standardized quality appraisal methods.

The COnsensus-based Standards for the selection of health Measurement INstruments (COSMIN) methodology offers a robust framework for evaluating health-related measurement instruments (Prinsen et al., 2018; Williams and Beovich, 2020). Comprising three core components—a risk-of-bias checklist, criteria for good measurement properties, and a modified GRADE approach—COSMIN has been applied to systematic reviews of multiple instruments within domains [e.g., motor assessments for youth (Hulteen et al., 2020)], single-instrument evaluations [e.g., Body Image Scale (Melissant et al., 2018)], and the Test of Gross Motor Development-3 (Zhu et al., 2025). Given the inconclusive evidence concerning SMS-II’s psychometric properties, we employed the COSMIN methodology to systematically evaluate its measurement properties, summarize extant research, and synthesize overall evidence quality.

## 2 Materials and methods

### 2.1 Literature search strategy

A systematic literature search was conducted in PubMed, Web of Science, EMBASE, PsycInfo, and CINAHL databases up to 20 March 2026 to identify studies evaluating the measurement properties of SMS-II. To optimize the search sensitivity for studies evaluating measurement properties, a comprehensive search strategy was employed, combining terms for the instrument and psychometric properties. Search terms for the instrument included (“Sport Motivation Scale-II” OR “SMS-II” OR “Sport motivation scale-ii” OR “revised sport motivation scale” OR “SMS-ii” OR “Sport Motivation Scale-2” OR “SMS-II” OR “Sport Motivation Scale 2nd” OR “Sport Motivation Scale-second edition”) AND terms related to measurement properties (reliability OR “internal consistency” OR “measurement error” OR “validity” OR “content validity” OR “face validity” OR “construct validity” OR “structural validity” OR “hypotheses testing” OR “cross-cultural validity” OR “criterion validity” OR responsiveness OR “measurement properties” OR “psychometric properties” OR “measurement property” OR “psychometric property” OR “divergent validity” OR “concurrent validity” OR “predictive validity”). This review was conducted in accordance with the Preferred Reporting Items for Systematic Reviews and Meta-Analyses (PRISMA) 2020 guidelines (Page et al., 2021). Full-text articles were retrieved from journal websites or acquired through institutional library services and external collaboration when necessary. The study protocol was registered with PROSPERO (CRD420251163217).

### 2.2 Inclusion and exclusion criteria

Studies were eligible for inclusion if they: (1) addressed SMS-II development or validation; and (2) evaluated at least one measurement property (content validity, structural validity, internal consistency, cross-cultural validity/measurement invariance, reliability, measurement error, criterion validity, hypothesis testing for construct validity, or responsiveness). The exclusion criteria were as follows: (1) studies using the SMS-II solely to assess intervention outcomes, (2) review or systematic review articles, and (3) non-peer-reviewed publications and those without full-text access.

### 2.3 Literature selection and data extraction

Literature search, study selection, and data extraction were conducted independently by two researchers (YZ and YY), with verification by two additional authors (HT and PY). Discrepancies were resolved through consultation with senior review authors (CZ and WG). Records were imported into EndNote for initial deduplication, followed by title and abstract screening. Full-text articles were then assessed against the eligibility criteria.

Extracted data elements included the first author, publication year, study population characteristics and recruitment sources, geographical region, sample size, participant age and sex distribution, SMS-II language version, evaluated measurement properties (content validity, structural validity, internal consistency, cross-cultural validity/measurement invariance, reliability, measurement error, criterion validity, hypothesis testing for construct validity, and responsiveness), and corresponding measurement property data.

### 2.4 Evaluation of the risk of bias and quality of evidence of included studies

Methodological quality was evaluated using the COSMIN risk-of-bias checklist (Mokkink et al., 2018), which comprises 10 domain-specific boxes (PROM development, content validity, structural validity, internal consistency, cross-cultural validity/measurement invariance, reliability, measurement error, criterion validity, hypothesis testing for construct validity, and responsiveness). Appropriate boxes were selected based on the measurement properties assessed in each study. Quality ratings were assigned at the item level as very good, adequate, doubtful, or inadequate, with overall study quality determined by the “worst-score principle” (lowest rating across items). Under this strict conservative approach, a study’s overall methodological rating for a specific measurement property is defined entirely by its lowest-scoring item; therefore, a single methodological flaw can substantially lower the study’s overall quality rating, regardless of high scores in other areas.

Evidence quality was synthesized using the modified GRADE approach adapted for COSMIN methodology (Mokkink et al., 2024), in which initial evidence was rated as high and then downgraded based on the risk of bias, inconsistency, indirectness, and imprecision; publication bias was excluded from this framework. Evidence quality was categorized as high, moderate, low, or very low according to the modified GRADE standards.

### 2.5 Overall rating of measurement property

The overall rating of each SMS-II measurement property was assessed against the COSMIN criteria for good measurement properties (Mokkink et al., 2024), encompassing nine domains: content validity, structural validity, internal consistency, cross-cultural validity/measurement invariance, reliability, measurement error, criterion validity, hypothesis testing for construct validity, and responsiveness (Supplementary Table S1). Individual items within each property were rated as sufficient (+), insufficient (−), or indeterminate (?), with overall property classifications designated as sufficient (+), insufficient (−), inconsistent (±), or indeterminate (?) (Supplementary Table S2). Inconsistent findings were analyzed in groups to explore the underlying sources of variation.

Hypothesis testing for construct validity requires the reviewer team to set the expected correlation thresholds in advance. Following the recommendations and illustrative examples provided in the COSMIN manual, and after achieving consensus through internal discussion among the review team regarding the theoretical overlap of the constructs, the hypotheses for this study were defined as follows. The comparator instrument measures a largely related construct (e.g., Global Motivation Scale, Task and Ego Orientation in Sport, Basic Needs Satisfaction in Sport, Satisfaction with Sport, Basic Psychological Needs in Exercise, and Passion) with expected correlations of *r* = 0.3–0.6. The comparator instrument measures a moderately related construct (e.g., Subjective Vitality, Satisfaction With Life, Interpersonal Behavior, Athlete Burnout, Flow State, Self-Confidence) with expected correlations of *r* = 0.2–0.5.

## 3 Results

### 3.1 Literature search results

**Figure 1:**
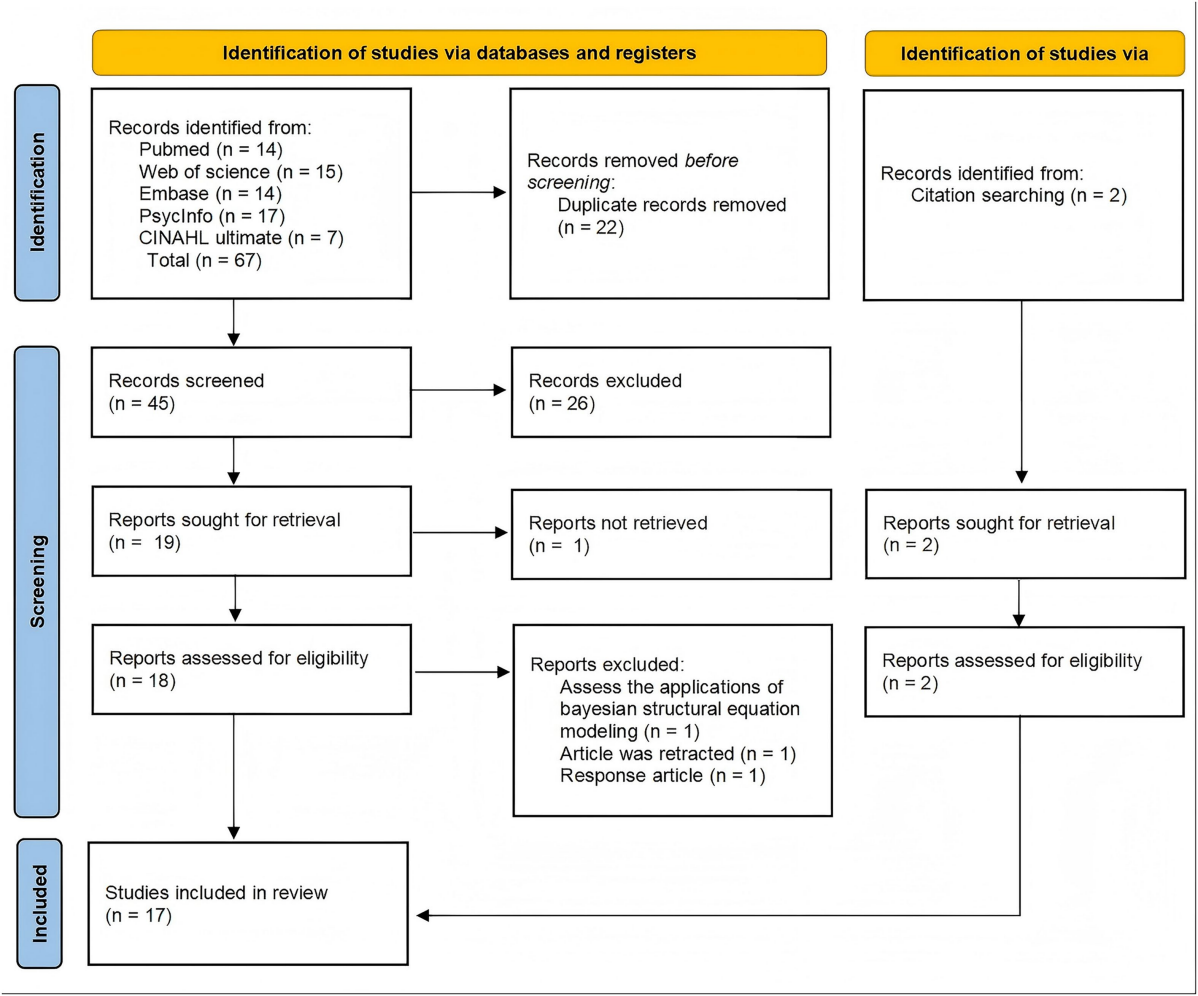
PRISMA flow diagram of article selection.

Database searches yielded 67 articles—14 from PubMed, 15 from Web of Science, 14 from EMBASE, 17 from PsycInfo, and 7 from CINAHL. The search, conducted up to 20 March 2026, imposed no restrictions on the publication date. After importation into EndNote, 22 duplicates were excluded. Title and abstract screening of the remaining 45 articles excluded 26 irrelevant articles (not meeting the inclusion criteria), leaving 19 articles for full-text review. One article was subsequently excluded owing to the unavailability of a complete manuscript (conference abstract), yielding 18 articles assessed for eligibility. Of these, three were excluded: one employed SMS-II to demonstrate Bayesian structural equation modeling applications (Stenling et al., 2015), one was retracted (Baaziz et al., 2023), and one constituted a commentary rather than primary research (Lonsdale et al., 2014). Additionally, a manual search of the reference lists of all full-text articles and relevant systematic reviews (snowballing technique) was conducted. Through this backward search process, two additional relevant articles meeting the inclusion criteria were identified and included in the final analysis. Ultimately, 17 articles met the inclusion criteria. The complete selection process is shown in Figure 1.

### 3.2 Characteristics of the included studies

The characteristics of the included studies are detailed in Table 1. These studies were primarily conducted in Europe (Paic et al., 2017; Viciana et al., 2017; Granero-Gallegos et al., 2018; Vallejo-Reyes et al., 2018; Pelletier et al., 2019; Yildiz et al., 2019; Jelínek et al., 2021; Rodrigues et al., 2021; Smohai et al., 2021; Jakšić et al., 2024; Pereira et al., 2024), Asia (Vallejo-Reyes et al., 2018; Chin et al., 2021), North America (Pelletier et al., 2013; Pineda-Espejel et al., 2016), and South America (Barreira et al., 2022; Nascimento Junior et al., 2025), with several studies including samples from multiple continents. Specifically, three studies were conducted in Spain (Viciana et al., 2017; Granero-Gallegos et al., 2018; Vallejo-Reyes et al., 2018), two in Portugal (Rodrigues et al., 2021; Pereira et al., 2024), two in Brazil (Barreira et al., 2022; Nascimento Junior et al., 2025), and two in Hungary (Paic et al., 2017; Smohai et al., 2021). The remaining literature is from France (Pelletier et al., 2019), Turkey (Yildiz et al., 2019), the Czech Republic (Jelínek et al., 2021), Serbia (Jakšić et al., 2024), China (Li et al., 2018), Malaysia (Chin et al., 2021), Canada (Pelletier et al., 2013), and Mexico (Pineda-Espejel et al., 2016). The participants were athletes (Pelletier et al., 2013, 2019; Pineda-Espejel et al., 2016; Paic et al., 2017; Viciana et al., 2017; Li et al., 2018; Vallejo-Reyes et al., 2018; Yildiz et al., 2019; Chin et al., 2021; Jelínek et al., 2021; Rodrigues et al., 2021; Smohai et al., 2021; Jakšić et al., 2024; Pereira et al., 2024; Nascimento Junior et al., 2025) and students (Granero-Gallegos et al., 2018; Barreira et al., 2022).

**Table 1:** Basic characteristics of the included articles.

Authors (year)	Population characteristics				Research characteristics of PACES		
	*N*	Age ( $\bar{x}$ ±SD)	Sex (M/F)	Studied population	Country/region	SMS-II language	Measurement properties
Pelletier et al. (2013)	Study 1: *n* = 412 Study 2: *n* = 290	Study 1: 40.44 ± 13.66 Study 2: 17.41 ± 1.77	Study 1: 104/218, unidentified = 90 Study 2: 113/177	Athletes	Canada	English	Structural validity Measurement invariance Convergent validity Internal consistency
Pineda-Espejel et al. (2016).	279	23.15 ± 5.58	143/131, unidentified = 5	High-performance athletes	Mexico	Spain	Structural validity Internal consistency
Paic et al. (2017)	500	21.16 ± 6.45	319/181	Athletes	Hungary	Hungarian	Structural validity Internal consistency Content validity
Viciana et al. (2017)	766	13.71 ± 1.30	503/263	Athletes	Spain	Spanish	Structural validity Internal consistency Measurement invariance
Granero-Gallegos et al. (2018)	1,055	Men: 13.87 ± 1.42 Women: 13.93 ± 1.37	519/536	Students	Spain	Spanish	Structural validity Internal consistency Measurement invariance Test–retest reliability Content validity
Li et al. (2018)	Study 1: *n* = 267 Study 2: *n* = 259	Study 1: 20.8 ± 1.39 Study 2: 20.5 ± 1.38	Study 1: 197/67, unidentified = 3 Study 2: 169/89, unidentified = 1	Athletes	China	Chinese	Structural validity Internal consistency Measurement invariance Convergent validity Content validity
Vallejo-Reyes et al. (2018)	221	22.00 ± 3.39	113/108	Athletes	Spain	Spanish	Content validity Internal consistency Structural validity
Pelletier et al. (2019)	Study 1: *n* = 247 Study 2: *n* = 259	Study 1: 17.07 ± 2.80 Study 2: 20.5 ± 1.38	Study 1: 127/110, unidentified = 10 Study 2: 89/170	Athletes	France	French	Structural validity Internal consistency Measurement invariance Convergent validity
Yildiz et al. (2019)	409	21.9 ± 4.3	241/168	Athletes	Turkey	Turkish	Structural validity Internal consistency Concurrent validity Convergent validity
Chin et al. (2021)	436	16.44 ± 1.22	299/137	Athletes	Malaysia	Malaysian	Content validity Structural validity Internal consistency
Jelínek et al. (2021)	243	16.42 ± 1.31	120/123	Adolescent athletes	Czech Republic	Czech	Structural validity Internal consistency Concurrent validity Content validity
Rodrigues et al. (2021)	1,148	18.45 ± 5.36	602/546	Athletes	Portugal	Portugal	Structural validity Measurement invariance Concurrent validity
Smohai et al. (2021)	1,197	11–67	599/598	Athletes	Hungary	Hungarian	Structural validity Internal consistency Convergent validity
Barreira et al. (2022)	Study 1: *n* = 304 Study 2: *n* = 441 Study 3: *n* = 310	Study 1: 21.8 ± 2.3 Study 2: 21.8 ± 3.9 Study 3: 21.9 ± 2.7	Study 1: 178/126 Study 2: 263/178 Study 3: 140/170	College students	Brazil	Brazilian	Structural validity Internal consistency
Jakšić et al. (2024)	365	13.79 ± 1.25	186/179	Young athletes	Serbia	Serbia	Structural validity Internal consistency Measurement invariance
Pereira et al. (2024)	239	14.39 ± 1.35	132/107	Adolescent athletes	Portugal	Portugal	Structural validity Measurement invariance Content validity Convergent validity
Nascimento Junior et al. (2025)	Study 1: *n* = 590 Study 2: *n* = 173	Study 1: 14.92 ± 1.68 Study 2: 12.96 ± 0.91	Study 1: 360/230 Study 2: 128/45	Athletes	Brazil	Brazilian	Structural validity Internal consistency Concurrent validity Content validity

M/F = male/female, y = years, $\bar{x}$ ±SD = Mean ± Standard deviation.

### 3.3 Synthesis of evidence for the measurement properties of SMS-II

The synthesis of the SMS-II measurement property evaluations and the quality of evidence for each property are shown in Table 2. The detailed evidence quality data are available in Supplementary Table S3.

**Table 2:** Summary of the findings.

Measurement property	Summary or pooled results		Overall rating	Quality of evidence
Content validity	Relevance: (Chin et al., 2021; Granero-Gallegos et al., 2018; Nascimento Junior et al., 2025; Pereira et al., 2024)		Sufficient (+)	**Moderate: multiple doubtful studies**
	Comprehensibility: (Chin et al., 2021; Granero-Gallegos et al., 2018; Jelínek et al., 2021; Li et al., 2018; Nascimento Junior et al., 2025; Pereira et al., 2024)		Sufficient (+)	**Moderate: multiple doubtful studies**
Structural validity	**Structural validity:** (Barreira et al., 2022; Chin et al., 2021; Granero-Gallegos et al., 2018; Jakšić et al., 2024; Jelínek et al., 2021; Li et al., 2018; Nascimento Junior et al., 2025; Paic et al., 2017; Pelletier et al., 2013, 2019; Pereira et al., 2024; Pineda-Espejel et al., 2016; Rodrigues et al., 2021; Vallejo-Reyes et al., 2018; Viciana et al., 2017; Yildiz et al., 2019)		**Qualitative summary: Sufficient (+)**	**Moderate or high**
	Six-factor structure:	Sufficient (+) (Pineda-Espejel et al., 2016; Paic et al., 2017; Viciana et al., 2017; Granero-Gallegos et al., 2018; Li et al., 2018; Vallejo-Reyes et al., 2018; Pelletier et al., 2019; Chin et al., 2021; Rodrigues et al., 2021; Smohai et al., 2021; Jakšić et al., 2024; Pereira et al., 2024; Nascimento Junior et al., 2025)	Sufficient (+) (76.5% supported)	Moderate: inconsistent findings
		Insufficient (−) (Barreira et al., 2022; Jelínek et al., 2021; Pelletier et al., 2013; Yildiz et al., 2019)		
	Two-factor structure: (Nascimento Junior et al., 2025; Viciana et al., 2017)		Insufficient (−)	High
	Three-factor structure: (Nascimento Junior et al., 2025; Viciana et al., 2017)		Insufficient (−)	High
	Five-factor structure: (Nascimento Junior et al., 2025; Viciana et al., 2017)		Sufficient (+)	High
	Four-factor structure: (Jelínek et al., 2021)		Insufficient (−)	High
Internal consistency	**Internal consistency:**		**Qualitative summary: inconsistent (±)**	**High**
	**Six-factor structure** (Pelletier et al., 2013, 2019; Pineda-Espejel et al., 2016; Paic et al., 2017; Viciana et al., 2017; Granero-Gallegos et al., 2018; Li et al., 2018; Vallejo-Reyes et al., 2018; Yildiz et al., 2019; Chin et al., 2021; Smohai et al., 2021; Barreira et al., 2022; Jakšić et al., 2024; Nascimento Junior et al., 2025)			
	Intrinsic: *α* = 0.55–0.89, (64.28% > 0.7)		Inconsistent (±)	High
	Integrated: *α* = 0.64–0.85, (71.43% > 0.7)		Inconsistent (±)	High
	Identified: *α* = 0.686–0.85, (85.71% > 0.7)		Sufficient (+)	Moderate: inconsistent findings
	Introjected: *α* = 0.39–0.79, (28.57% > 0.7)		Inconsistent (±)	High
	External: *α* = 0.536–0.85, (64.28% > 0.7)		Inconsistent (±)	High
	Amotivated: *α* = 0.526–0.81, (50.00% > 0.7)		Inconsistent (±)	High
	**Five-factor structure** (Viciana et al., 2017)			
	Intrinsic: *α* = 0.737		Sufficient (+)	High
	Integrated: *α* = 0.744		Sufficient (+)	High
	Identified: *α* = 0.700		Insufficient (−)	High
	External: *α* = 0.655		Insufficient (−)	High
	Amotivated: *α* = 0.635		Insufficient (−)	High
Reliability	**Test–retest reliability:** (Chin et al., 2021; Granero-Gallegos et al., 2018; Li et al., 2018)		**Qualitative summary:** **Sufficient (+)**	**High**
	Intrinsic: ICC = 0.80–0.90		Sufficient (+)	High
	Integrated: ICC = 0.82–0.84		Sufficient (+)	High
	Identified: ICC = 0.53–0.84, (66.67% > 0.7)		Inconsistent (±)	High
	Introjected: ICC = 0.70–0.84		Sufficient (+)	High
	External: ICC = 0.73–084		Sufficient (+)	High
	Amotivated: ICC = 0.84–0.90		Sufficient (+)	High
Cross-cultural validity/measurement invariance	**Cross-cultural validity/measurement invariance**		**Qualitative summary: Sufficient (+)**	**High**
	Cross-cultural validity: (Pelletier et al., 2019) No important differences (ΔCFI < 0.01)		Sufficient (+)	High
	Across sex groups: (Granero-Gallegos et al., 2018; Li et al., 2018; Pelletier et al., 2013, 2019; Pereira et al., 2024; Viciana et al., 2017) No important differences (ΔCFI < 0.01)		Sufficient (+)	High
	Across age groups: (Li et al., 2018; Pelletier et al., 2013) No important differences (ΔCFI < 0.01)		Sufficient (+)	High
	Across sports-federated groups: (Viciana et al., 2017) No important differences (ΔCFI < 0.01)		Sufficient (+)	High
Hypothesis testing for construct validity	**Hypothesis testing for construct validity:** (Jelínek et al., 2021; Li et al., 2018; Nascimento Junior et al., 2025; Pelletier et al., 2013, 2019; Rodrigues et al., 2021; Yildiz et al., 2019)		**Qualitative summary: Sufficient (+)**	**High**
	SMS-II and GMS: (Pelletier et al., 2013)			
	Intrinsic:		Sufficient (+)	High
	Integrated:		Sufficient (+)	High
	Identified:		Sufficient (+)	High
	Introjected:		Sufficient (+)	High
	External:		Sufficient (+)	High
	Amotivated:		Sufficient (+)	High
	SMS-II Identified and SWLS: (Pelletier et al., 2013, 2019; Li et al., 2018; Yildiz et al., 2019; Jelínek et al., 2021; Smohai et al., 2021)		Sufficient (+)	High
	SMS-II Intrinsic and SV: (Li et al., 2018; Pelletier et al., 2013; Yildiz et al., 2019)		Sufficient (+)	High
	SMS-II Intrinsic and TEOSQ Task: (Pelletier et al., 2013, 2019; Yildiz et al., 2019)		Sufficient (+)	High
	SMS-II Intrinsic and IBS Coach autonomy: (Pelletier et al., 2013, 2019)		Sufficient (+)	High
	SMS-II Amotivated and ABQ: (Li et al., 2018)		Sufficient (+)	High
	SMS-II Intrinsic and SWSS (Pelletier et al., 2019)		Sufficient (+)	High
	SMS-II Amotivated and PS (Nascimento Junior et al., 2025)		Sufficient (+)	High
	SMS-II Intrinsic and Need satisfaction BNSSS (Pelletier et al., 2019)		Sufficient (+)	High
	SMS-II External and Need thwarting BNSSS (Pelletier et al., 2019)		Sufficient (+)	High
	SMS-II Integrated and BPNES autonomy (Rodrigues et al., 2021)		Sufficient (+)	High
	SMS-II Integrated and CSAI-2 Self-Confidence (Smohai et al., 2021)		Sufficient (+)	High
	SMS-II Integrated and PPL-FSQ Flow State (Smohai et al., 2021)		Sufficient (+)	High

*α* = Cronbach’s alpha values, ICC = Intraclass correlation coefficient, ΔCFI = Change in Comparative Fit Index, GMS = Global Motivation Scale, SWLS = Satisfaction with life scale, SV = Subjective vitality scale, TEOSQ = Task and ego orientation in sport questionnaire, IBS = Interpersonal Behavior Scale, ABQ = Athlete burnout questionnaire, SWSS = Satisfaction with Sport Scale, BNSSS = Basic needs satisfaction in sport, BPNES = Basic psychological needs exercise scale, CSAI-2 = Competitive State Anxiety Inventory 2, PPL-FSQ = the Flow State Questionnaire of the Positive Psychology Lab. Bold text indicates overall assessment of the following content.

#### 3.3.1 Content validity

Among the 17 included studies, 6 evaluated the content validity of SMS-II using the COSMIN methodology (Granero-Gallegos et al., 2018; Li et al., 2018; Chin et al., 2021; Jelínek et al., 2021; Pereira et al., 2024; Nascimento Junior et al., 2025). These assessments, conducted via expert consultation and athlete feedback, yielded sufficient ratings for relevance and comprehensibility with moderate-quality evidence. However, comprehensiveness was not evaluated across all studies, preventing the determination of overall content validity and qualitative synthesis (Table 2).

#### 3.3.2 Structural validity

All 17 included articles assessed the structural validity of the SMS-II (Pelletier et al., 2013, 2019; Pineda-Espejel et al., 2016; Paic et al., 2017; Viciana et al., 2017; Granero-Gallegos et al., 2018; Li et al., 2018; Vallejo-Reyes et al., 2018; Yildiz et al., 2019; Chin et al., 2021; Jelínek et al., 2021; Rodrigues et al., 2021; Smohai et al., 2021; Barreira et al., 2022; Jakšić et al., 2024; Pereira et al., 2024; Nascimento Junior et al., 2025).

Each article examined the six-factor structure of SMS-II (Pelletier et al., 2013, 2019; Pineda-Espejel et al., 2016; Paic et al., 2017; Viciana et al., 2017; Granero-Gallegos et al., 2018; Li et al., 2018; Vallejo-Reyes et al., 2018; Yildiz et al., 2019; Chin et al., 2021; Jelínek et al., 2021; Rodrigues et al., 2021; Smohai et al., 2021; Barreira et al., 2022; Jakšić et al., 2024; Pereira et al., 2024; Nascimento Junior et al., 2025), with 13 of which supporting structural validity was sufficient (Pineda-Espejel et al., 2016; Paic et al., 2017; Viciana et al., 2017; Granero-Gallegos et al., 2018; Li et al., 2018; Vallejo-Reyes et al., 2018; Pelletier et al., 2019; Chin et al., 2021; Rodrigues et al., 2021; Smohai et al., 2021; Jakšić et al., 2024; Pereira et al., 2024; Nascimento Junior et al., 2025), and 4 finding it supporting insufficient structural validity (Pelletier et al., 2013; Yildiz et al., 2019; Jelínek et al., 2021; Barreira et al., 2022). Given that 76.5% of the articles supported sufficient structural validity, the overall rating was sufficient. The evidence quality was moderate due to inconsistent findings. Three studies examined the two-factor, three-factor, four-factor, and five-factor structures of SMS-II (Viciana et al., 2017; Jelínek et al., 2021; Nascimento Junior et al., 2025). The five-factor structure demonstrated sufficient structural validity, whereas the two-factor and three-factor structures showed insufficient structural validity. The evidence quality was high, and the risk of bias of all studies was judged as very good.

#### 3.3.3 Internal consistency

Thirteen of the 17 included articles assessed the internal consistency of the SMS-II (Pelletier et al., 2013, 2019; Pineda-Espejel et al., 2016; Paic et al., 2017; Viciana et al., 2017; Granero-Gallegos et al., 2018; Li et al., 2018; Vallejo-Reyes et al., 2018; Yildiz et al., 2019; Chin et al., 2021; Barreira et al., 2022; Jakšić et al., 2024; Nascimento Junior et al., 2025).

Fourteen studies examined the internal consistency of each subscale under the six-factor structure of the SMS-II (Pelletier et al., 2013, 2019; Pineda-Espejel et al., 2016; Paic et al., 2017; Viciana et al., 2017; Granero-Gallegos et al., 2018; Li et al., 2018; Vallejo-Reyes et al., 2018; Yildiz et al., 2019; Chin et al., 2021; Smohai et al., 2021; Barreira et al., 2022; Jakšić et al., 2024; Nascimento Junior et al., 2025). Cronbach’s alpha values for each subscale were 0.55–0.89 (Intrinsic, 64.28% > 0.7), 0.64–0.85 (Integrated, 71.43% > 0.7), 0.686–0.85 (Identified, 85.71% > 0.7), 0.39–0.79 (Introjected, 28.57% > 0.7), 0.536–0.85 (External, 64.28% > 0.7) and 0.526–0.81 (Amotivated, 50.00% > 0.7). The internal consistency of the Identified subscale was rated as sufficient (85.71% supported), and the evidence quality was moderate due to inconsistent findings. However, while the other subscales were rated as insufficient, the evidence quality was high because the risk of bias in all studies was judged as very good.

One study also examined the internal consistency of each subscale under the five-factor structure of the SMS-II (Viciana et al., 2017). Cronbach’s alpha values for the subscales were 0.737 (Intrinsic), 0.744 (Integrated), 0.700 (Identified), 0.655 (External), and 0.635 (Amotivated). The internal consistency of the Intrinsic and Integrated subscales was rated as sufficient, whereas the Identified, External, and Amotivated subscales were rated as insufficient. The evidence quality was high.

#### 3.3.4 Cross-cultural validity/measurement invariance

Six articles assessed the measurement invariance (across sex, age, sport-federated, and non-federated) or cross-cultural validity of the SMS-II using multi-group confirmatory factor analysis (MCFA). In these studies, overall model fit and invariance were primarily evaluated using the Comparative Fit Index (CFI), Tucker–Lewis Index (TLI), and Root Mean Square Error of Approximation (RMSEA). In accordance with the COSMIN guidelines, measurement invariance was considered sufficient if the change in CFI (ΔCFI) between nested models (e.g., configural, metric, scalar) was less than 0.01. Applying this objective criterion, six studies reported a ΔCFI < 0.01, indicating sufficient measurement invariance across sex groups. The quality of evidence for these studies was judged to be high (Pelletier et al., 2013, 2019; Viciana et al., 2017; Granero-Gallegos et al., 2018; Li et al., 2018; Pereira et al., 2024). Similarly, two studies demonstrated sufficient measurement invariance across age groups (ΔCFI < 0.01). The quality of evidence for these studies was judged to be high (Pelletier et al., 2013; Li et al., 2018). One study confirmed sufficient measurement invariance between sport-federated and non-federated participants (ΔCFI < 0.01) with high-quality evidence (Viciana et al., 2017). One study assessed the cross-cultural validity of the SMS-II and found no significant differences (ΔCFI < 0.01), suggesting sufficient cross-cultural validity. The quality of evidence in this study was judged to be high (Pelletier et al., 2019).

In summary, the findings suggest that the SMS-II has sufficient cross-cultural validity and measurement invariance across sex, age, and sport-federated groups, supported by high-quality evidence.

#### 3.3.5 Reliability

Three studies examined the test–retest reliability of the SMS-II by calculating Intraclass correlation coefficients (ICCs) over specified time intervals, utilizing either a one-week interval (Granero-Gallegos et al., 2018; Li et al., 2018) or a four-week interval (Chin et al., 2021) between measurements. The ICCs for each subscale were 0.80–0.90 (Intrinsic), 0.82–0.84 (Integrated), 0.53–0.84 (Identified, 66.67% > 0.7), 0.70–0.84 (Introjected), 0.73–0.84 (External), and 0.84–0.90 (Amotivated). The test–retest reliability of the Identified subscale was rated as inconsistent (66.67% > 0.7), and the evidence quality was high. The other subscales were rated as sufficient with high-quality evidence.

#### 3.3.6 Hypothesis testing for construct validity

Eight articles evaluated hypothesis testing for construct validity of the SMS-II (Pelletier et al., 2013, 2019; Li et al., 2018; Yildiz et al., 2019; Jelínek et al., 2021; Rodrigues et al., 2021; Smohai et al., 2021; Nascimento Junior et al., 2025). These studies assessed the construct validity of the SMS-II by examining the correlation between the SMS-II subscale and similar domain measurement instruments. These measurement instruments were the Global Motivation Scale (GMS) (Pelletier et al., 2013) Satisfaction with life scale (SWLS) (Pelletier et al., 2013, 2019; Li et al., 2018; Yildiz et al., 2019; Jelínek et al., 2021; Smohai et al., 2021), Subjective vitality scale (SV) (Pelletier et al., 2013; Li et al., 2018; Yildiz et al., 2019), Task and ego orientation in sport questionnaire-Task (TEOSQ) (Pelletier et al., 2013, 2019; Yildiz et al., 2019), Interpersonal Behavior Scale-CA (IBS) (Pelletier et al., 2013), Athlete Burnout Questionnaire (ABQ) (Li et al., 2018), Satisfaction with Sport Scale (SWSS) (Pelletier et al., 2019), the Passion Scale (PS) (Nascimento Junior et al., 2025), Interpersonal Behavior Scale (IBS) (Pelletier et al., 2013, 2019), Need satisfaction-Basic needs satisfaction in sport (BNSSS) (Pelletier et al., 2019), Need thwarting-Basic needs satisfaction in sport (BNSSS) (Pelletier et al., 2019), Basic Psychological Needs in Exercise Scale (BPNES)-autonomy (Rodrigues et al., 2021), Competitive State Anxiety Inventory-2 (CSAI-2) (Smohai et al., 2021), and the Flow State Questionnaire of the Positive Psychology Lab (PPL-FSQ) (Smohai et al., 2021) (Table 2).

One study assessed the convergent validity of the SMS-II subscales with the GMS subscales (Pelletier et al., 2013). The overall rating results showed that the convergent validity of all the subscales (Integrated, Identified, Introjected, Intrinsic, Amotivated, and External) was sufficient (*r* > 0.5). The quality of evidence was high. Five studies assessed the convergent validity of the SMS-II Intrinsic subscale with SV (Pelletier et al., 2013; Li et al., 2018; Yildiz et al., 2019), TEOSQ Task (Pelletier et al., 2013, 2019; Yildiz et al., 2019), IBS Coach autonomy (Pelletier et al., 2013, 2019), SWSS (Pelletier et al., 2019), and Need satisfaction BNSSS (Pelletier et al., 2019). Six studies assessed the convergent validity of the SMS-II Identified subscale with the SWLS (Pelletier et al., 2013, 2019; Li et al., 2018; Yildiz et al., 2019; Jelínek et al., 2021; Smohai et al., 2021). Two studies assessed the convergent validity of the SMS-II Amotivated subscale with the ABQ (Li et al., 2018) and the PS (Nascimento Junior et al., 2025). Two studies assessed the convergent validity of the SMS-II External subscale with PS (Nascimento Junior et al., 2025) and Need thwarting BNSSS (Pelletier et al., 2019). Three studies assessed the convergent validity of the SMS-II Integrated subscale with BPNES autonomy (Rodrigues et al., 2021), CSAI-2 Self-Confidence (Smohai et al., 2021), and PPL-FSQ Flow State (Smohai et al., 2021). The overall rating results showed that the convergent validity was sufficient with high-quality evidence.

## 4 Discussion

To the best of our knowledge, this is the first systematic review to use the COSMIN methodology to assess the measurement properties of the SMS-II. In this study, we evaluated the different measurement properties of the SMS-II reported in 17 articles. Our findings were as follows: the SMS-II had sufficient content validity (relevance and comprehensibility, moderate-quality evidence), six-factor structural validity (moderate-quality evidence), five-factor structural validity (high-quality evidence), cross-cultural validity/measurement invariance (across sex, age, sports, high-quality evidence), internal consistency under the six-factor structure (Identified subscale, moderate-quality evidence), internal consistency under the five-factor structure (Integrated and Intrinsic subscale, high-quality evidence), test–retest reliability (Intrinsic, Integrated, Introjected, External and Amotivated subscale, high-quality evidence), and hypothesis testing for construct validity (high-quality evidence). Two-factor, three-factor, and four-factor structural validity and internal consistency under the five-factor structure (Identified, External, and Amotivated subscales) were rated as insufficient with high-quality evidence. Other measurement properties were assessed as inconsistent with high-quality evidence.

According to the COSMIN manual, content validity refers to whether the content of an instrument appropriately reflects the construct to be measured, which is the most important measurement property of the instrument (Mokkink et al., 2024). Content validity includes three aspects: relevance, comprehensiveness, and comprehensibility. The content validity of SMS-II demonstrated that relevance (Granero-Gallegos et al., 2018; Chin et al., 2021; Pereira et al., 2024; Nascimento Junior et al., 2025) and comprehensibility (Granero-Gallegos et al., 2018; Li et al., 2018; Chin et al., 2021; Jelínek et al., 2021; Pereira et al., 2024; Nascimento Junior et al., 2025) were sufficient, with moderate quality of evidence. However, no study has evaluated the comprehensiveness of the SMS-II items. The aspect of comprehensiveness ensures that no key facets of the construct are missing (Terwee et al., 2018). The comprehensiveness of the SMS-II was not evaluated in any of the included studies. The implication of this critical gap is that we cannot definitively conclude whether the SMS-II captures the absolute entirety of an athlete’s motivational experience. While the instrument aligns well with the foundational SDT continuum, it remains possible that certain contextual, modern, or culturally specific facets of sport motivation are missing from the current item pool. To resolve this indeterminate rating and address this gap, it is imperative that future studies employ rigorous qualitative methodologies. Specifically, researchers should conduct in-depth cognitive interviews and focus groups with diverse athlete populations to verify the comprehensiveness of the scale and ensure that no critical motivational dimensions have been overlooked. Furthermore, the application of the conservative “worst-score principle” in our risk of bias assessment revealed a consistent methodological weakness across the literature that must be addressed: the inadequate reporting of qualitative procedures. Most studies downgraded in their content validity assessment suffered from a lack of detailed descriptions of the interview processes conducted with expert panels or target subjects (athletes). To resolve the indeterminate ratings and address this gap, it is imperative that future research not only employs rigorous qualitative methodologies—such as in-depth cognitive interviewing and focus groups with diverse athlete populations—but also transparently reports these procedures. Future validation studies must explicitly detail their qualitative protocols, including participant selection criteria, interview guides, and analytical frameworks, to achieve high methodological quality and ensure no critical motivational dimensions have been overlooked.

Structural validity pertains to how well the scores of a scale capture the dimensional characteristics of the underlying construct it is intended to measure (Mokkink et al., 2024). According to the SDT, the highest form of self-determination is intrinsic motivation, which corresponds to extrinsic motivation (Deci and Ryan, 2000). Extrinsic motivation is composed of four internalization stages: External regulation, Introjected regulation, Identified regulation, and Integrated regulation. In terms of increasing degree of internalization, External regulation > Introjected regulation > Identified regulation > Integrated regulation. Pelletier et al. applied the SDT to add the Amotivated subscale; thus, SMS-II has six dimensions (Intrinsic, Integrated, Identified, Introjected, External, and Amotivated) (Pelletier et al., 2013). Our evaluation results show that 13 studies (76.5%) support that the six-factor structural validity of SMS-II is sufficient, but there are still 4 studies that consider it insufficient. According to the COSMIN manual, 75% of the studies recognize it as sufficient, and the overall assessment can be rated as sufficient; however, the quality of evidence needs to be downgraded (Mokkink et al., 2024). Therefore, we believe that moderate-quality evidence indicates that the six-factor structural validity of the SMS-II is sufficient. Consistent with our findings, Amaro et al.’s review also confirmed the adequate six-factor structural validity of the SMS-II (Amaro et al., 2025). In addition, two studies removed the Introjected dimension in SMS-II and examined the five-factor structural validity of SMS-II. The results showed that the five-factor structural validity of the SMS-II is sufficient with high-quality evidence. Given the weight of the evidence, with 13 studies (76.5%) supporting the six-factor model and its direct alignment with the complete SDT framework, we definitively recommend that future researchers prioritize the theoretical breadth of the six-factor structure. While the five-factor model (which removes the Introjected dimension) demonstrated sufficient validity with high-quality evidence, relying on this truncated model sacrifices the theoretical integrity for statistical parsimony. Abandoning the introjected regulation prevents researchers from fully capturing the complex internal pressures athletes face. Therefore, rather than defaulting to the five-factor model to achieve a better statistical fit, we strongly advise researchers to retain the six-factor structure and manage its statistical instability by aggregating problematic adjacent subscales into composite scores (e.g., controlled motivation). The five-factor model should only be utilized in highly specific contexts, where strict statistical parsimony is an absolute priority over capturing the full motivational continuum. We recommend that future research continue to evaluate the structural validity of the SMS-II, specifically investigating the performance of the Introjected dimension to better understand the contextual factors that occasionally necessitate a five-factor solution.

Internal consistency refers to the degree of interrelatedness among items of the same subscale (Mokkink et al., 2024). Cronbach’s alpha values exceeding 0.7 are typically regarded as indicative of sufficient internal consistency. A total of 14 studies evaluated the internal consistency of the SMS-II under a six-factor structure (Pelletier et al., 2013, 2019; Pineda-Espejel et al., 2016; Paic et al., 2017; Viciana et al., 2017; Granero-Gallegos et al., 2018; Li et al., 2018; Vallejo-Reyes et al., 2018; Yildiz et al., 2019; Chin et al., 2021; Smohai et al., 2021; Barreira et al., 2022; Jakšić et al., 2024; Nascimento Junior et al., 2025), and one study evaluated the internal consistency of the SMS-II under a five-factor structure (Viciana et al., 2017). The results showed that only the Identified subscale satisfied 75% of Cronbach’s alpha greater than 0.7 and was evaluated as sufficient, while the other subscales were evaluated as inconsistent findings under the six-factor structure. It is critical to note the practical implications of the particularly low internal consistency observed in some studies, such as the alpha of 0.39 for the Introjected subscale. Such low values indicate a severe lack of interrelatedness among the items. However, evaluating these psychometric inconsistencies through the theoretical lens of Self-Determination Theory (SDT) provides a crucial explanatory context. SDT posits that behavioral regulations exist along a continuum characterized by a simplex pattern where adjacent motivational constructs (e.g., external and introjected regulation) share inherent conceptual similarities. This theoretical overlap frequently manifests statistically as lower internal consistency and factor cross-loading. For instance, recurring issues with the Introjected and External subscales may not merely reflect poor item wording, but rather the genuine cognitive difficulty athletes face in distinguishing between internal pressures (introjection) and external contingencies (external regulation) within a highly pressured sports environment. Consequently, utilizing subscales with such poor internal consistency—whether due to item flaws or theoretical overlap—poses a significant risk of generating invalid data and drawing erroneous conclusions in sports practice. To address subscales demonstrating poor internal consistency (i.e., Cronbach’s alpha < 0.7), we recommend a dual strategy that combines immediate statistical solutions with long-term measurement refinement. In the short term, researchers should move beyond classical test theory by employing a higher-order model with composite scoring. Specifically, a six-factor first-order structure (external, introjected, identified, integrated, intrinsic, and amotivation) should be specified to load onto a second-order factor representing the degree of self-determination. For practical applications, composite scores should be calculated by combining external and introjected regulations into controlled motivation and identified, integrated, and intrinsic regulations into autonomous motivation. The Relative Autonomy Index (RAI) can then be derived as a weighted difference between autonomous and controlled motivation. This approach acknowledges the theoretical overlap between adjacent constructs—consistent with SDT’s simplex pattern—while providing statistically robust and practically interpretable indices that mitigate the risks associated with low-reliability subscales. In the long term, we recommend employing Item Response Theory (IRT) or Rasch analysis to pinpoint precise item-level deficiencies within problematic subscales. Furthermore, for items identified as problematic, targeted semantic revisions should be conducted—potentially guided by cognitive interviewing with athletes—to ensure that the wording unambiguously captures the intended motivational construct. These revised items should be re-evaluated for interrelatedness before implementation in future research. This dual approach ensures both immediate analytical rigor and progressive improvement of the measurement instrument.

Test–retest reliability refers to the degree to which scores remain consistent over time (Mokkink et al., 2024). Our results showed that the test–retest reliability of the SMS-II was sufficient for all subscales, except for the Identified subscale. Only one study reported the ICC value for the Identified subscale, which was <0.7, and we did not identify any risk of bias for this study. Therefore, we recommend further research to evaluate the test–retest reliability of the Identified subscale.

As outlined in the COSMIN manual, cross-cultural validity refers to “the extent to which the performance of items on translated or culturally adapted measurement instruments accurately reflects the performance of the items in the original version of those instruments” (Mokkink et al., 2024). Measurement invariance refers to the consistency of a test’s scores across different groups (e.g., sex and age) (Mokkink et al., 2024). Regarding cross-cultural validity and measurement invariance, six studies provided high-quality evidence demonstrating sufficient invariance across sex, age, and sport types (Granero-Gallegos et al., 2018; Li et al., 2018; Pelletier et al., 2013, 2019; Pereira et al., 2024; Viciana et al., 2017). Establishing strict measurement invariance confirms that the SMS-II operates consistently across diverse athletic demographics. Consequently, researchers can be confident that any observed differences in motivation scores—whether between male and female athletes, different age cohorts, or federated versus non-federated competitors—reflect genuine variations in the latent motivational constructs rather than methodological artifacts or measurement bias. This makes the SMS-II a uniquely robust and highly recommended instrument for comparative cross-sectional and longitudinal research in sport psychology. While older or less rigorously evaluated scales often struggle with structural equivalence across diverse samples (thereby risking the generation of artificial group differences), the SMS-II provides a highly reliable foundation for conducting multi-group comparative analyses across broad athletic populations.

Responsiveness refers to a scale’s ability to detect meaningful changes in a construct measured over time (Mokkink et al., 2024). Measurement error refers to the systematic and random error of a participant’s score, which is not attributed to true changes in the construct to be measured (Mokkink et al., 2024). We did not find any studies that assessed the responsiveness or measurement error of the SMS-II. Finally, we recommend using the methods recommended by the COSMIN manual to conduct in-depth research on the responsiveness and measurement error of the SMS-II. To effectively evaluate responsiveness, future studies should employ robust longitudinal designs, such as pre- and post-intervention measurements over at least an eight-week period. These methodological approaches are crucial for evaluating the SMS-II’s ability to detect meaningful changes in motivational constructs over time and for ensuring measurement accuracy.

According to the COSMIN manual’s recommendations for measurement instrument selection, a tool is recommended if there is high-quality evidence that all its measurement properties are sufficient. However, this is not recommended if there is high-quality evidence that one or more properties are insufficient. In other cases, a recommendation for more high-quality evidence has been provided (Mokkink et al., 2024). In our study, the two-factor, three-factor, and four-factor structural validities of the SMS-II were rated as insufficient, but this only indicates that these specific factor models are not suitable for the SMS-II. This does not mean that the structural validity of the SMS-II as a measurement property is insufficient. The six-factor model may be more appropriate for the SMS-II, and its structural validity was rated as sufficient. Similarly, the insufficient rating for the internal consistency of the three subscales (Identified, External, and Amotivated) under the five-factor structure might suggest that the six-factor model is more suitable for the SMS-II. Therefore, we can conclude that none of the core measurement properties of the SMS-II itself were ultimately rated as insufficient. The SMS-II can be used, but more high-quality research is needed to demonstrate that all of its currently inconsistent measurement properties are sufficient.

This study has the following limitations and future perspectives. First, this study did not exclude non-English and non-Chinese articles, and software-assisted translation was used during the process of reading and data extraction, which may have led to the omission of data or information. Second, the data and information on content validity extracted in this study were obtained from the methods sections of the articles, not as primary outcomes, which led to inaccuracies. Finally, we recommend using the methods outlined in the COSMIN manual to conduct in-depth research on the responsiveness and measurement error of the SMS-II. These properties are crucial for evaluating the SMS-II’s ability to detect meaningful changes over time and for ensuring measurement accuracy. By expanding the empirical research on these measurement properties, future studies can consolidate the application value of the SMS-II across different contexts and populations, thereby enhancing its applicability and reliability in sport training and educational environments.

## 5 Conclusion

The assessment of the SMS-II using the COSMIN methodology demonstrated that there is moderate- to high-quality evidence that content validity (relevance and comprehensibility), six-factor structural validity, five-factor structural validity, cross-cultural validity/measurement invariance, internal consistency under the six-factor structure (Identified subscale), internal consistency under the five-factor structure (Integrated and Intrinsic subscale), test–retest reliability (except Identified subscale), and hypothesis testing for construct validity are sufficient. Two-factor, three-factor, and four-factor structural validity and internal consistency under the five-factor structure (Identified, External, and Amotivated subscales) were rated as insufficient. Other measurement properties were assessed as inconsistent with high-quality evidence. We conclude that the SMS-II can be utilized in sport and exercise contexts, provided that researchers and practitioners apply specific operational safeguards. For structural guidance, the six-factor model remains preferable owing to its comprehensive alignment with the Self-Determination Theory. However, confidence in the subscales varies significantly. Subscales measuring autonomous forms of motivation (i.e., Intrinsic, Integrated, and Identified regulation) generally demonstrate better psychometric stability and can be used with higher confidence, although caution remains warranted regarding the temporal stability of the Identified subscale. Conversely, subscales measuring controlled forms of motivation (Introjected, External, and Amotivation) frequently demonstrated insufficient internal consistency. To mitigate these limitations, we strongly recommend that practitioners avoid relying solely on isolated scores from the problematic subscales. Instead, researchers should combine them into broader, theoretically sound composite scores to improve overall reliability and ensure valid interpretations. Further high-quality research, particularly utilizing modern psychometric approaches and robust longitudinal designs, is imperative to refine these inconsistent measurement properties.

## Data Availability

The datasets presented in this study can be found in online repositories. The names of the repository/repositories and accession number(s) can be found in the article/Supplementary material.
