# Supplementary material for: Assessing the reliability and validity of the sport motivation scale-II using the COSMIN methodology—a systematic review
Source: Front Psychol. 2026 May 21;17:1807146. doi: 10.3389/fpsyg.2026.1807146 (PMC13233221; doi:10.3389/fpsyg.2026.1807146)
Supplement: Supplementary file 1 [file Table_1.DOCX]

Supplementary Material

# Supplementary Figures and Tables

**Table S1. COSMIN Definitions of Measurement Properties**

| **Term** | | | **Definition** |
| --- | --- | --- | --- |
| **Domain** | **Measurement**  **property** | **Measurement**  **property aspect** |  |
| Reliability |  |  | The degree to which the measurement is free from measurement error |
| Reliability  (extended  definition) |  |  | The extent to which scores for patients who have not changed are the same for repeated measurement under several conditions: e.g. using different sets of items from the scale (internal consistency); over time (test-retest); by different persons on the same occasion (inter-rater); or by the same persons (i.e. raters or responders) on different occasions (intra-rater) |
|  | Internal  Consistency |  | The degree of the interrelatedness among the items |
|  | Reliability |  | The proportion of the total variance in the measurements which is due to “true”a differences between patients |
|  | Measurement  Error |  | The systematic and random error of a patient’s score that is not attributed to “true”a  changes in the construct to be measured |
| Validity |  |  | The degree to which the scale measures the construct(s) it purports to measure (depressive symptom severity) |
|  | Content Validity |  | The degree to which the content of the scale is an adequate reflection of the construct to be measured |
|  |  | Face Validity | The degree to which (the items of) the scale indeed looks as though they are an adequate reflection of the construct to be measured |
|  | Construct  Validity |  | The degree to which the scores of the scale are consistent with hypotheses (for instance with regard to internal relationships, relationships to scores of other instruments, or differences between relevant groups) based on the assumption that the  PDMS-2 validly measures the construct to be measured |
|  |  | Structural Validity | The degree to which the scores of the scale are an adequate reflection of the dimensionality of the construct to be measured |
|  |  | Hypothesis-Testing | Item construct validity |
|  |  | Cross-cultural  validity/measurement invariance | The degree to which the performance of the items on a translated or culturally adapted scale are an adequate reflection of the performance of the items of the original version of the scale. |
| Responsiveness | Responsiveness |  | The ability of the scale to detect change over time in the construct to be measured |

**Table S2. COSMIN Criteria for Assessing the Measurement Properties**

| **Measurement**  **property** | **Rating** | **Criteria for rating** |
| --- | --- | --- |
| Structural Validity | + | Classical Test Theory: Confirmatory Factor Analysis: Comparative Fit Index or Tucker-Lewis Index or comparable measure >0.95 OR Root Mean Square Error of Approximation <0.06 or Standardized Root  Mean Residuals <0.082 |
|  | ? | Classical Test Theory: Not all information for + reported |
|  | - | Criteria for + not met |
| Internal consistency | + | At least low evidence for sufficient structural validity AND Cronbach's alpha(s) ≥ 0.70 for each unidimensional scale or subscale |
|  | ? | Criteria for “At least low evidence for sufficient structural validity” not met |
|  | - | At least low evidence for sufficient structural validity AND Cronbach’s alpha(s) < 0.70 for each unidimensional scale or subscale |
| Cross-cultural  validity/measurement  invariance | + | No important differences found between group factors (such as age, gender, language) in multiple group factor analysis OR no important differential item functioning for group factors (McFadden's R2 < 0.02) |
|  | ? | No multiple group factor analysis OR differential item functioning analysis performed |
|  | - | Important differences between group factors OR differential item functioning was found |
| Reliability | + | Intraclass correlation coefficient or weighted Kappa ≥ 0.70 |
|  | ? | Intraclass correlation coefficient or weighted Kappa not reported |
|  | - | Intraclass correlation coefficient or weighted Kappa < 0.70 |
| Measurement error | + | Smallest detectable change or limits of agreement < minimal important change |
|  | ? | Minimal important change not defined |
|  | - | Smallest Detectable Change or Limits of Agreement > Minimal Important Change |
| Criterion validity | + | Correlation with gold standard ≥ 0.70 OR Area Under the Curve ≥ 0.70 |
|  | ? | Not all information for + reported |
|  | - | Correlation with gold standard < 0.70 OR Area Under the Curve < 0.70 |
| Hypothesis testing for  construct validity | + | The result is in accordance with the hypothesis |
|  | ? | No hypothesis defined (by the review team) |
|  | - | The result is not in accordance with the hypothesis |
| Responsiveness | + | The result is in accordance with the hypothesis OR area under the curve ≥ 0.70 |
|  | ? | No hypothesis defined (by the review team) |
|  | - | The result is not in accordance with the hypothesis OR area under the curve < 0.70 |

Note:”+” = sufficient; “-” = insufficient; “?” = indeterminate

**Table S3. Levels of Evidence for the Measurement Properties of the SMS-2**

| **Measurement**  **properties** | | **Methodological risk of**  **bias** | **Inconsistency** | **Imprecision** | **Indirectness of**  **evidence** | **GRADE level**  **(total number of**  **evidence**  **downgrades)** |
| --- | --- | --- | --- | --- | --- | --- |
| Content validity | | YES (-1)  multiple  doubtful studies | NO | NO | NO | Moderate (-1) |
| Structural validity | Six-factor structure: | NO  All studies of adequate  methodological quality | YES(-1)  inconsistent results | NO  N> 100 | NO | Moderate (-1) |
|  | Two-factor structure: | NO  Two studies of adequate  methodological quality | NO | NO  N> 100 | NO | High |
|  | Three-factor structure: | NO  Two studies of adequate  methodological quality | NO | NO  N> 100 | NO | High |
|  | Five-factor structure: | NO  Two studies of adequate  methodological quality | NO | NO  N> 100 | NO | High |
|  | Four-factor structure: | NO  One study of adequate  methodological quality | NO | NO  N> 100 | NO | High |
| Internal consistency under the six-factor structure | Intrinsic | NO  Thirteen studies of adequate  methodological quality | NO | NO  N> 100 | NO | High |
|  | Integrated: | NO  Thirteen studies of adequate  methodological quality | NO | NO  N> 100 | NO | High |
|  | Identified: | NO  Thirteen studies of adequate  methodological quality | YES(-1)  inconsistent results | NO  N> 100 | NO | Moderate (-1) |
|  | Introjected: | NO  Thirteen studies of adequate  methodological quality | NO | NO  N> 100 | NO | High |
|  | External: | NO  Thirteen studies of adequate  methodological quality | NO | NO  N> 100 | NO | High |
|  | Amotivated | NO  Thirteen studies of adequate  methodological quality | NO | NO  N> 100 | NO | High |
| Internal consistency under the five-factor structure | Intrinsic | NO  One study of adequate  methodological quality | NO | NO  N> 100 | NO | High |
|  | Integrated: | NO  One study of adequate  methodological quality | NO | NO  N> 100 | NO | High |
|  | Identified: | NO  One study of adequate  methodological quality | NO | NO  N> 100 | NO | High |
|  | External: | NO  One study of adequate  methodological quality | NO | NO  N> 100 | NO | High |
|  | Amotivated | NO  One study of adequate  methodological quality | NO | NO  N> 100 | NO | High |
| Cross-cultural validity\  measurement invariance | | NO  Six studies of adequate  methodological quality | NO | NO  N> 100 | NO | High |
| Test-retest Reliability | | NO  Three studies of adequate  methodological quality | NO | NO  N> 100 | NO | High |
| Hypothesis testing for construct validity | | NO  All studies of adequate  methodological quality | NO | NO  N> 100 | NO | High |

Note: N/A = Not applicable, i.e., no included studies assessed that particular measurement property.
